# Autonomous Collision Avoidance Using MPC with LQR-Based Weight Transformation

**DOI:** 10.3390/s21134296

**Published:** 2021-06-23

**Authors:** Shayan Taherian, Kaushik Halder, Shilp Dixit, Saber Fallah

**Affiliations:** Department of Mechanical Engineering Sciences, Connected Autonomous Vehicle Lab (CAV-Lab), University of Surrey, Guildford GU2 7XH, UK; s.taherian@surrey.ac.uk (S.T.); k.halder@surrey.ac.uk (K.H.); s.dixit@surrey.ac.uk (S.D.)

**Keywords:** trajectory planning, MPC, LQR, LQT, inverse optimal control, collision avoidance

## Abstract

Model predictive control (MPC) is a multi-objective control technique that can handle system constraints. However, the performance of an MPC controller highly relies on a proper prioritization weight for each objective, which highlights the need for a precise weight tuning technique. In this paper, we propose an analytical tuning technique by matching the MPC controller performance with the performance of a linear quadratic regulator (LQR) controller. The proposed methodology derives the transformation of a LQR weighting matrix with a fixed weighting factor using a discrete algebraic Riccati equation (DARE) and designs an MPC controller using the idea of a discrete time linear quadratic tracking problem (LQT) in the presence of constraints. The proposed methodology ensures optimal performance between unconstrained MPC and LQR controllers and provides a sub-optimal solution while the constraints are active during transient operations. The resulting MPC behaves as the discrete time LQR by selecting an appropriate weighting matrix in the MPC control problem and ensures the asymptotic stability of the system. In this paper, the effectiveness of the proposed technique is investigated in the application of a novel vehicle collision avoidance system that is designed in the form of linear inequality constraints within MPC. The simulation results confirm the potency of the proposed MPC control technique in performing a safe, feasible and collision-free path while respecting the inputs, states and collision avoidance constraints.

## 1. Introduction

MPC has been recognised as one of the most powerful multi-objective optimal control techniques for a wide range of applications with its ability to formulate the system constraints as a finite-horizon constrained optimisation problem. MPC performance is highly reliant on the weights on each objective but the challenge is that there is no systematic procedure to select the weights to assure the best performance of MPC. Currently, suitable weight selection is a time consuming procedure that requires a large number of trial and error selections and many computer simulations [1,2,3]. The complexity increases with an increase in the number of control objectives [4]. Hence, the necessity of developing a precise tuning procedure for MPC weights has arisen. This has been investigated by previous research works using different techniques [5,6]. For example, [6] presented a solution for selecting weights in order to match the MPC as an LQR-based linear controller. In this work, an LMI-based LQR approach was chosen by recasting the DARE in the LMI form to calculate the weighting matrices of an unconstrained MPC. Although this approach was found as a solution for MPC control parameters, the cost function was limited to have zero cross state-space terms, such that only a few controllers could be matched. In [7], the matching controller, i.e., the task of matching the MPC performance with the performance of a favorite controller, has been addressed by presenting a systematic method to tune the parameters of generalized unconstrained MPC for SISO systems [8]. In this approach the tuning parameters were calculated based on the desired closed-loop transfer function in order to relate the tuning parameters to the desired specification of the system. However, in this algorithm, a higher control horizon leads to a set of inequality bilinear constraints in the unknown elements, which cannot be solved using convex optimisation for linear MPC. In [9], the authors proposed an analytical tuning method for the Dynamic Matrix Control (DMC) algorithm. DMC is one of the primary versions of MPC that utilizes the step response of the plant, which can be approximated by the first-order plus delay time (FOPDT) model. The proposed analytical method offers several advantages, such as low computational cost and the conservative behaviour of the control system in chemical plants. However, this approach fails to demonstrate the effectiveness of applying quadratic programming solvers or additional adaptive mechanisms within the controller architecture. Other tuning strategies have been investigated by researchers using intelligent learning algorithms [10,11]. In [12,13], the authors implemented neural networks and fuzzy-based decision making to tune the MPC parameters, without using the reference system model. The authors of [1] proposed an automated strategy for an MPC-based motion cueing algorithm optimized using a multi-objective genetic algorithm. This strategy found suitable MPC weights, aiming to demonstrate the superiority of the proposed tuning strategy over empirical tuning methods without the prior knowledge of the plant model. The general idea behind auto-tuning using learning and neural network algorithms is that the tuning parameters are updated along with the optimisation algorithm, and thus the solution is always set to the optimal value. Moreover, it is not required to have a great amount of knowledge about the system model to initialize the tuning procedure. However, it is needed to operate two optimisation procedures per time step (optimisation required by intelligent algorithms and additional optimisation required by the MPC controller) that may require overhead computational time. In [14], the authors proposed a full review of tuning strategies for different MPC formulations, such as dynamic matrix control, generalized predictive control and state-space representation of an MPC controller. Moreover, a review on a few autotuning strategies is available in this work. The authors mentioned that these strategies benefit from not having full knowledge of the model of the system to initialize the tuning procedure. However, the major disadvantage of the autotuning strategy is the computational overhead, which has to perform two optimizations compared to offline tuning strategies. In [15], the authors proposed an evolutionary algorithm using a non-dominated sorting genetic algorithm for a multi-objective optimisation problem within an MPC framework. This method offers simple procedure to handle MPC constraints. Moreover, this algorithm suggests a flexible representation of the decision variables that expedite the controller performance. However, this algorithm suffers from computation overhead, as it requires two optimisations per time step.

In this paper, the inverse optimal control problem [16,17,18] of a discrete time linear quadratic regulator (DLQR) is used for designing an MPC controller to perform collision avoidance manoeuvres by selecting appropriate weighting matrices. In general, the DLQR weights (Q,R) are used to solve the discrete algebraic Riccati equation (DARE) for discrete time systems in order to find a solution (*P*) that is a real symmetric positive definite matrix. Then, using *P*, an optimal state feedback control gain can be obtained from the DLQR control law. However, these weighting matrices obtained from DLQR can also be used to compute the controller of unconstrained MPC [6]. Therefore, using the aforementioned concept, the objective of this paper is to design the weighting matrix *Q* of the DLQR in such a way that *Q* can be used within the MPC framework, while assuring asymptotical stability of the system [19]. DLQR and unconstrained MPC can be related through DARE, but using the weighting matrix obtained from the inverse optimal control problem of DLQR in MPC design cannot ensure solving the reference tracking problem. To overcome this problem, we recast DLQR into a discrete time linear quadratic tracking (DLQT) problem [20] to design the weighting matrix *Q*, which can be used for MPC design to ensure solving the reference tracking problem. In order to do this, the dynamics of the vehicle and Ackerman pole-placement criteria are considered in the formulation of *Q* selection through DLQT, which will be used in MPC design for a trajectory planning task. In order to highlight the practical use of this analytical tuning technique, the proposed technique is implemented to generate safe, admissible and collision-free trajectories for autonomous vehicle collision avoidance systems. In this work, a collision avoidance system is formulated as a finite horizon optimisation problem with vehicle dynamics, while the collision avoidance constraints are formulated within the MPC algorithm. However, collision avoidance constraints are generally non-convex in the context of trajectory planning, which limits the feasibility and uniqueness of the solution of the optimisation problem. To tackle this problem, researchers provide solutions such as convexification [21], changing the reference frame [22,23] and affine linear collision avoidance constraints [24]. In this paper the collision avoidance constraints are formulated in the form of linear affine constraints to perform trajectory planning manoeuvres on a curve path. Moreover, in the formulation of the collision avoidance constraints, the longitudinal position of the vehicle is excluded, allowing the designers to reduce the state dimensions of the system. This is advantageous, as removing a state from the system model helps in lowering the computational overhead for solving the optimisation problem in MPC. The effectiveness of the entire framework for autonomous collision avoidance system on a curved road is validated in a co-simulation platform where a high-fidelity vehicle model is simulated in IPG-CarMaker while the proposed MPC design is implemented in MATLAB/Simulink.

The contributions of this paper are summarised as follows: *(i)* Design of an analytical tuning technique to perform a collision-free trajectory for autonomous vehicles, and *(ii)* development of a mathematical framework to design convex constraints in order to perform collision avoidance manoeuvres on a curved path.

## 2. Discrete Time Optimal Controller Design

In this section, the tuning strategy for designing the MPC controller with the appropriate selection of the weighting matrix *Q* is described. To design the tuning strategy, a discrete time inverse optimal control problem is formulated using a linear time invariant (LTI) system:

(1) $$\bar{x}\left(k+1\right)=\bar{A_{d}}\bar{x}\left(k\right)+\bar{B_{d}}u\left(k\right)$$

The objective of this strategy is to design the *Q* matrix from a discrete time algebraic Ricatti equation, which will ensure almost equal optimality between the DLQR and the unconstrained MPC control problem. It is noted that in this design the weighting factor *R* is chosen as a fixed value as in [19]. Moreover, the weighting matrix *Q* obtained from the inverse DLQR problem should be designed in such a way that the closed-loop poles are placed in the desired location within the unit circle by satisfying user-defined specifications using Ackerman’s pole placement formula. It is noted that this design is a procedure for finding the MPC weighting matrix *Q* by obtaining the sub-optimal solution from the DLQR controller in the presence of the constraints.

In the following subsections a general discrete infinite linear quadratic regulator problem is briefly overviewed and then a recast in DLQR as a discrete linear quadratic tracking (DLQT) problem. Next, with the concept of the inverse DLQT control problem, a strategy for designing an analytical approach to select the *Q* matrix for the MPC control design will be discussed.

### 2.1. Discrete Time Linear Quadratic Regulator (DLQR)

To design the discrete time optimal controller, the standard infinite horizon quadratic cost needs to be minimized, corresponding to the discrete time system model (1):

(2) $$J=\sum_{k=0}^{\infty }\bar{x}\left(k\right)Q^{T}\bar{x}\left(k\right)+u\left(k\right)R^{T}u\left(k\right)$$

where $Q=Q^{T}\geq 0$ and $R=R^{T}=[\begin{array}{cc} 10 & 0\\ 0 & 9\times 10^{-2} \end{array}]>0$. The solution of the DARE can be obtained to minimize the performance index (2) and is given by:

(3) $$P=Q+{\bar{A}}_{d}^{T}P{\bar{A}}_{d}-{\bar{A}}_{d}^{T}P{\bar{B}}_{d}{\left(R+{\bar{B}}_{d}^{T}P{\bar{B}}_{d}\right)}^{-1}{\bar{B}}_{d}^{T}P{\bar{A}}_{d}$$

The discrete time optimal controller gain $\bar{K}$ can be achieved using the matrix $P=P^{T}=\left[\begin{array}{ccc} P_{11} & P_{12} & P_{13}\\ P_{21} & P_{22} & P_{23}\\ P_{31} & P_{32} & P_{33} \end{array}\right]>0$ calculated from (3) to minimizes the cost function (2). The controller gain $\bar{K}$ can be written as:

(4) $$\bar{K}={\left(R+{\bar{B}}_{d}^{T}P{\bar{B}}_{d}\right)}^{-1}{\bar{B}}_{d}^{T}P{\bar{A}}_{d}$$

which gives the control law:

(5) $$u\left(k\right)=-\bar{K}\bar{x}\left(k\right)$$

### 2.2. Discrete Time Linear Quadratic Tracking (DLQT)

In this paper, an augmented system is presented based on the system dynamic (1) and the reference trajectory. The objective for the DLQT problem is to find the optimal control law in such a way that the designed controller can be ensured to track the reference trajectory for the linear system (1) within the predefined finite horizon performance index. The augmented system becomes:

(6) $$\begin{array}{c} X_{k+1}=\left[\begin{array}{c} {\bar{x}}_{k+1}\\ r_{k+1} \end{array}\right]=\underset{\hat{A}}{\underset{︸}{\left[\begin{array}{cc} \bar{A_{d}} & 0\\ 0 & F \end{array}\right]}}\underset{X_{k}}{\underset{︸}{\left[\begin{array}{c} {\bar{x}}_{k}\\ r_{k} \end{array}\right]}}+\underset{\hat{B}}{\underset{︸}{\left[\begin{array}{c} {\bar{B}}_{d}\\ 0 \end{array}\right]}}u\left(k\right) \end{array}$$

where *F* is assumed to be Hurwitz. This strategy is adopted from [20], and for the brevity of the paper, an interested reader can refer to this technical paper for calculation of the DLQT algebraic Riccati equation:

(7) $$Q_{1}-P_{1}+{\hat{A}}^{T}P_{1}\hat{A}-{\hat{A}}^{T}P_{1}\hat{B}{\left(R+{\hat{B}}^{T}P_{1}\hat{B}\right)}^{-1}{\hat{B}}^{T}P_{1}\hat{A}=0$$

where $Q_{1}$ is the weighting matrix for the augmented system (1):

(8) $$Q_{1}=\left[\begin{array}{cc} Q & 0\\ 0 & Q \end{array}\right]$$

and $P_{1}$ is the solution of the algebraic Riccati equation:

(9) $$P_{1}=P_{1}^{T}>0$$

Then the control law can be written as:

(10) $$u\left(k\right)=-{\left(R+{\hat{B}}^{T}P_{1}\hat{B}\right)}^{-1}{\hat{B}}^{T}P_{1}\hat{A}X\left(k\right)$$

where the value of the controller gain is:

(11) $$\hat{K}={\left(R+{\hat{B}}^{T}P_{1}\hat{B}\right)}^{-1}{\hat{B}}^{T}P_{1}\hat{A}$$

For the reference tracking problem using MPC for the system (6), the Ricatti Equation (7) holds. The *Q* matrix obtained from the DLQR problem can be used for the DLQT control problem as shown in (8). Therefore, *Q* can be obtained by solving the inverse DLQR optimal control problem to use it in the reference tracking problem from the DLQT approach. Hence, we can use the *Q* matrix obtained from the inverse DLQR approach in the MPC control problem, since the MPC reference tracking problem is equivalent to the DLQT control problem. The following section describes the system model that has been used for the MPC tuning strategy.

## 3. System Model

In this paper, a kinematic vehicle model is used to capture the vehicle model in MPC [25]. This model assumes no slip between the tyre and the road, and is found to be a suitable model for trajectory planning for the application of collision avoidance manoeuvres [26,27]. Under the assumption of small angles approximation [28] the vehicle bicycle model is:

(12) $$\dot{\xi }=v_{x}$$

(13) $$\dot{y}=v_{x}\psi +\frac{l_{r}}{l_{f}+l_{r}}v_{x}\delta_{f}$$

(14) $${\dot{v}}_{x}=a_{x}$$

(15) $$\dot{\psi }=\frac{v_{x}\delta_{f}}{l_{f}+l_{r}}$$

where ξ and *y* are the longitudinal and lateral displacement of the vehicle in inertial coordinates, respectively, ψ is the inertial heading angle of the vehicle, $v_{x}$ is the longitudinal velocity of the vehicle, $l_{f}$ is the distance of the front axle from the centre of gravity (C.G), and $l_{r}$ is the distance of the rear axle from C.G. The control inputs are the steering angle $\delta_{f}$ and longitudinal acceleration $a_{x}$. For a given nominal velocity $v_{x,nom}$, the system in (12), (13), (14), (15) can be expressed using an LTI system using the compact notation given below:

(16) $$\dot{x}=Ax+Bu$$

where $x≜{\left[\xi ,y,v_{x},\psi \right]}^{T}\in X\subseteq R^{4}$ is the state vector and $u≜{\left[\delta_{f},a_{x}\right]}^{T}\in U\subseteq R^{2}$ is the input vector with X and U are polyhedron regions used as state and input constraints, respectively. The structure of the system matrix *A* and input matrix *B* are as follows:

(17) $$\left[\begin{array}{c} \dot{\xi }\\ \dot{y}\\ {\dot{v}}_{x}\\ \dot{\psi } \end{array}\right]=\underset{A}{\underset{︸}{\left[\begin{array}{cccc} 0 & 0 & 1 & 0\\ 0 & 0 & 0 & v_{x,\mathrm{nom}}\\ 0 & 0 & 0 & 0\\ 0 & 0 & 0 & 0 \end{array}\right]}}\left[\begin{array}{c} \xi \\ y\\ v_{x}\\ \psi \end{array}\right]+\underset{B}{\underset{︸}{\left[\begin{array}{cc} 0 & 0\\ \frac{v_{x,\mathrm{nom}}l_{r}}{\left(l_{r}+l_{f}\right)} & 0\\ 0 & 1\\ \frac{v_{x,\mathrm{nom}}}{\left(l_{r}+l_{f}\right)} & 0 \end{array}\right]}}\left[\begin{array}{c} \delta \\ a_{x} \end{array}\right]\;$$

where $v_{x,nom}$ is the nominal longitudinal velocity of the vehicle, which is chosen in the same way as the initial speed of the vehicle [3]. Then system (16) is descretised with a sampling time $t_{s}$ to obtain the linear time invariant discrete system shown below:

(18) $$x\left(k+1\right)=A_{d}x\left(k\right)+B_{d}u\left(k\right)$$

where $A_{d}=e^{At_{s}}=\left[\begin{array}{cccc} 1 & 0 & t_{s} & 0\\ 0 & 1 & 0 & v_{x,\mathrm{nom}}t_{s}\\ 0 & 0 & 1 & 0\\ 0 & 0 & 0 & 1 \end{array}\right]$ and $B_{d}=Bt_{s}=\left[\begin{array}{cc} 0 & 0\\ \frac{v_{x,\mathrm{nom}}l_{r}t_{s}}{L} & 0\\ 0 & t_{s}\\ \frac{v_{x,\mathrm{nom}}t_{s}}{L} & 0 \end{array}\right]$

As the dynamics of the state ξ in system (17) depends only on *v*, it is possible to further simplify the system for trajectory generation:

(19) $$\bar{x}\left(k+1\right)=\bar{A_{d}}\bar{x}\left(k\right)+\bar{B_{d}}u\left(k\right)$$

where $\bar{x}={\left[y,v,\psi \right]}^{T}$ is the system states, $u=[\delta_{f},a_{x}]$ is the system inputs, and matrices ${\bar{A}}_{d}$ and ${\bar{B}}_{d}$ are obtained by extracting the appropriate rows and columns of $A_{d}$ and $B_{d}$, respectively, in (17).

## 4. Selection of Weighting Matrix Q for MPC Design

In order to obtain the appropriate *Q* matrix, for designing an MPC-based reference tracking controller, we have chosen $Q=diag(Q_{11},Q_{22},Q_{33})$ for system (19) and the optimal control gain $\bar{K}$ with a fixed weighting factor *R*. For the reference tracking problem, MPC can be recast as a DLQT problem as described in Section 2.2. Therefore, it can be inferred that the weighting matrix *Q* obtained from the inverse DLQT problem will provide almost the same controller gain for MPC, which is described in the following Theorem.

**Theorem** **1.** *MPC will ensure the reference tracking for system (19), guaranteeing the sub-optimality of the DLQT control problem by satisfying (2)–(5) with $Q_{1}^{T}=Q_{1}\geq 0$, $R^{T}=R>0$ and $P_{1}^{T}=P_{1}>0$, where $Q_{1}=diag\left(Q,Q\right)$, $R_{1}=diag\left(R,R\right)$ and the controller gains $\hat{K}=diag\left(\bar{K},\bar{K}\right)$ with $Q=diag(Q_{11},Q_{22},Q_{33})$, and $P_{1}=diag\left(P,P\right)$ where P will be as (3) and $\bar{K}=\left[\begin{array}{ccc} k_{1} & k_{2} & k_{3}\\ k_{4} & k_{5} & k_{6} \end{array}\right]$, respectively.*

**Proof.**  Equation (3) can be recast as a DLQR problem:

(20) $$Q=P-{\bar{A}}_{d}^{T}P{\bar{A}}_{d}+{\bar{A}}_{d}^{T}P{\bar{B}}_{d}{\left(R+{\bar{B}}_{d}^{T}P{\bar{B}}_{d}\right)}^{-1}{\bar{B}}_{d}^{T}P{\bar{A}}_{d}$$

where $P=P^{T}=\left[\begin{array}{ccc} P_{11} & P_{12} & P_{13}\\ P_{21} & P_{22} & P_{23}\\ P_{31} & P_{32} & P_{33} \end{array}\right]>0$, $R=\left[\begin{array}{cc} R_{1} & 0\\ 0 & R_{2} \end{array}\right]>0$ and

$Q=Q^{T}=\left[\begin{array}{ccc} Q_{11} & Q_{12} & Q_{13}\\ Q_{21} & Q_{22} & Q_{23}\\ Q_{31} & Q_{32} & Q_{33} \end{array}\right]\geq 0$. The corresponding control law (4) can be written as:

(21) $$\bar{K}=\left[\begin{array}{ccc} k_{1} & k_{2} & k_{3}\\ k_{4} & k_{5} & k_{6} \end{array}\right]={\left(R+{\bar{B}}_{d}^{T}P{\bar{B}}_{d}\right)}^{-1}{\bar{B}}_{d}^{T}P{\bar{A}}_{d}$$

Since the input $\bar{B_{d}}$ matrix in (19) includes only three non-zero elements, the three components of controller gains $\left(k_{2},k_{4},k_{6}\right)$ will be zero, and the rest of the gains will be non-zero elements. The following section describes the system model required for designing the weighting matrix within the MPC controller.    □

Now, using (19), (21) and the matrices (*P*,*Q*,*R*) in (20) yields the diagonal and off-diagonal elements of the *Q* matrix as shown in (25)–(27) and

(22) $$Q_{12}=-k_{2}\left[P_{11}\frac{vl_{r}t_{s}}{L}+P_{13}\frac{vt_{s}}{L}\right]-P_{12}t_{s}k_{5}$$

(23) $$Q_{13}=vt_{s}P_{11}-\left[k_{3}\left[P_{11}\frac{vl_{r}t_{s}}{L}+P_{13}\frac{vt_{s}}{L}\right]+P_{12}t_{s}k_{6}\right]$$

(24) $$\begin{array}{c} Q_{32}=vt_{s}P_{12}-[[\frac{vl_{r}}{L}t_{s}\left(vt_{s}P_{11}+P_{13}\right)\\ +\frac{v}{L}t_{s}\left(vt_{s}P_{13}+P_{33}\right)]k_{2}+\left(vt_{s}P_{12}+P_{23}\right)k_{5}] \end{array}$$

respectively. Since matrix *Q* is diagonal, all of the off-diagonal elements are equal to zero, i.e., $Q_{12}=Q_{13}=Q_{32}=0$. Therefore, using these conditions and gains $k_{2}=k_{4}=k_{6}=0$, the elements of the *P* matrix can be obtained, i.e., (22) yields (32). Using (32) in (23) yields (29), and using (32) in (24) results in (33). Now, using the elements (32), (33), (29) and the *R*, $B_{d}$ matrices in (21), Equations (34)–(36) will hold. Finally, by solving (34)–(36), Equations (28), (30) and (31) can be obtained. It is noted that the gain matrix $\bar{K}$ needs to be specified such that the closed-loop poles of the system (18) would be inside the unit circle. In this paper, Ackerman’s pole placement formula is used to obtain the gains ($\bar{K}$) by specifying the closed-loop pole location inside the unit circle. This method solves the solution of the Riccati equation to generate optimal weights using an LQR controller. The generated gains are then used in the MPC controller, which inherits all of the properties and optimal solutions from the LQR controller when MPC constraints are not active. However, when the constraints are active, the solution of the Riccati equation in LQR provides a sub-optimal solution for MPC, as it satisfies the constraints of the Quadratic Programming (QP) problem indirectly. In other words, the set of states $\bar{x}\left(k\right)$ where the matching occurs is the polyhedron, where the unconstrained optimizer (i.e., unconstrained MPC, therefore the DLQR controller) satisfies the constraints of the QP problem [6].

(25) $$\begin{array}{cc} Q_{11} & =\left(P_{11}\frac{vl_{r}}{l_{r}+l_{f}}t_{s}+P_{13}\frac{v}{l_{r}+l_{f}}t_{s}\right)k_{1} \end{array}$$

(26) $$\begin{array}{cc} Q_{22} & =P_{22}t_{s}k_{5} \end{array}$$

(27) $$\begin{array}{cc} Q_{33} & =-vt_{s}P_{13}+\left[\frac{vl_{r}}{l_{r}+l_{f}}t_{s}\left(vt_{s}P_{11}+P_{13}\right)+\frac{v}{l_{r}+l_{f}}t_{s}\left(vt_{s}P_{13}+P_{33}\right)\right] \end{array}$$

(28) $$\begin{array}{cc} P_{13} & =\frac{-\frac{R_{1}k_{1}vt_{s}}{L}\left[\frac{k_{3}vt_{s}}{L}+\left(1-\frac{vt_{s}}{L}\right)\right]}{\frac{v^{4}t_{s}^{4}k_{1}}{L^{3}}\left[\frac{vl_{r}t_{s}k_{3}}{L}-l_{r}-vt_{s}\right]\left(1-\frac{l_{r}k_{3}}{L}\right)-\frac{v^{3}t_{s}^{3}}{L^{2}}\left[\left(1-\frac{vl_{r}t_{s}k_{1}}{L}\right)\left(1-\frac{vt_{s}}{L}\right)\left(1-\frac{l_{r}k_{3}}{L}\right)\right]+\frac{v^{2}l_{r}t_{s}^{2}}{L}\left(\frac{l_{r}k_{3}}{L}-1\right)\frac{v^{3}t_{s}^{3}k_{1}k_{3}}{L^{3}}} \end{array}$$

(29) $$\begin{array}{cc} P_{11} & =-\frac{P_{13}\frac{v}{L}t_{s}k_{3}}{\frac{vl_{r}}{L}t_{s}k_{3}-vt_{s}} \end{array}$$

(30) $$\begin{array}{cc} P_{33} & =\frac{P_{11}\left[\frac{vt_{s}l_{r}}{L}-\frac{k_{1}v^{2}l_{r}^{2}t_{s}^{2}}{L^{2}}\right]-k_{1}R_{1}+P_{13}\left[\frac{vt_{s}}{L}-\frac{v^{2}l_{r}t_{s}^{2}k_{1}}{L^{2}}\right]}{\frac{k_{1}v^{2}ts^{2}}{L^{2}}} \end{array}$$

(31) $$\begin{array}{cc} P_{22} & =\frac{R_{2}k_{5}}{ts-ts^{2}k_{5}} \end{array}$$

(32) $$\begin{array}{cc} P_{12} & =0 \end{array}$$

(33) $$\begin{array}{cc} P_{23} & =0 \end{array}$$

(34) $$\begin{array}{cc} k_{1} & =\frac{\frac{P_{11}vl_{r}t_{s}}{L}+\frac{P_{13}vt_{s}}{L}}{R_{1}+\frac{v^{2}l_{r}^{2}t_{s}^{2}P_{11}}{L^{2}}+\frac{\left[\frac{P_{13}vl_{r}t_{s}}{L}+\frac{P_{33}vt_{s}}{L}\right]vt_{s}}{L}} \end{array}$$

(35) $$\begin{array}{cc} k_{3} & =\frac{\frac{P_{11}v^{2}l_{r}t_{s}}{L}+P_{13}\frac{\left[vt_{s}P_{13}+P_{33}\right]vt_{s}}{L}}{R_{1}+\frac{P_{11}v^{2}t_{s}^{2}}{L^{2}}+\frac{\left[\frac{P_{13}vt_{s}}{L}+\frac{P_{3}3vt_{s}}{L}\right]vt_{s}}{L}} \end{array}$$

(36) $$\begin{array}{cc} k_{5} & =\frac{P_{22}t_{s}}{R_{2}+P_{22}t_{s}^{2}} \end{array}$$

(37) $$\begin{array}{cc} k_{2} & =k_{4}=k_{6}=0 \end{array}$$

## 5. Constraint Design

In this section, the mathematical framework for computing the collision avoidance constraints that are used to plan trajectories on curved roads is presented. In the absence of traffic vehicles, a convex region is designed to ensure that the planned vehicle trajectories remain within the boundaries of the road. On the other hand, in the presence of obstacles, a convex region is designed to ensure that the planned trajectories remain within the road boundaries and maintain a safe distance from the obstacle. A detailed explanation of the aforementioned constraints designs is presented in the following subsections.

### 5.1. Road Boundary Constraints

**Figure 1:**
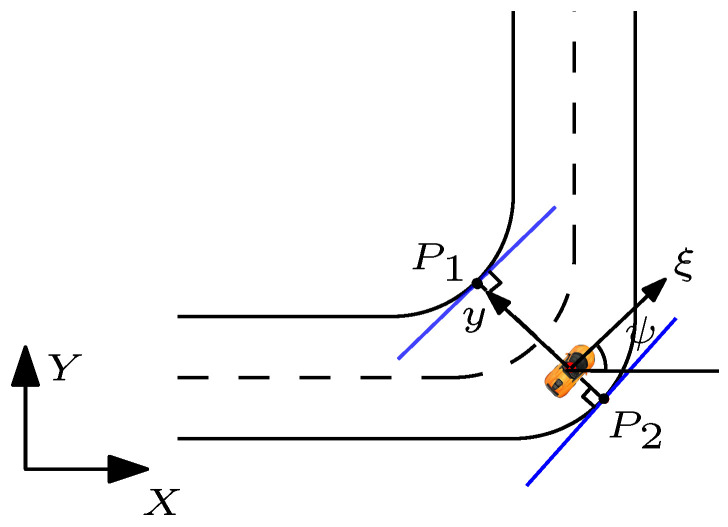
The hyperplanes (blue lines) indicate the constraints for optimization. The points on the borders are the projection of the centre line of the subject vehicle on the borders.

Using the edges of a curved road as constraints within the MPC framework results in non-convex constraints, which is not suitable for a Quadratic Programming (QP) framework [29]. Consequently, boundary constraints are designed by assuming that the road segment in the immediate vicinity of the subject vehicle is part of a straight road (Figure 1). The edges of the virtual straight road are obtained by calculating the points $P_{1}$ and $P_{2}$ in Figure 1. These virtual straight lines can be constructed using the generated points $P_{1}$ and $P_{2}$, followed by the slope equal to the heading angle of the upper and lower road boundaries. Moreover, the equation of these lines can be formulated in the form of linear inequality constraints, which can ensure that the planned trajectories lie within the edges of this virtual road. The point $P_{1}$ is located at the intersection of the left (outer) road edge with an imaginary line passing through the subject vehicle’s centre of gravity (C.G) and perpendicular to the subject vehicle’s longitudinal axis. The equations of the outer lane boundary and the intersected line are:

(38) $$y_{outer}=f\left(x_{outer}\right)$$

(39) $$a_{outer}\xi +b_{outer}y+c_{outer}=0$$

where (38) is the equation of the outer lane boundary, (39) is the equation of the intersected line perpendicular to the outer lane boundary, and the parameters $a_{outer},b_{outer}$ and $c_{outer}$ in Equation (39) are the coefficients of the intersected line. The line passing through $P_{1}$ with a slope equal to the reference path heading angle $\psi_{ref}$ forms the outer edge (left line) of the virtual straight road (blue line in Figure 1) with the following equation:

(40) $$m_{outer}\xi -y+P_{1y}-m_{outer}P_{1x}<0$$

where $m_{outer}$ is the slope of the equation of the outer edge road and $\left(P_{1x},P_{1y}\right)$ are coordinate positions of point $P_{1}$. From Equation (12) it is evident that the evolution of ξ depends on the longitudinal velocity of the vehicle. Therefore, the predicted longitudinal position ξ can be estimated using the initial position $\xi_{0}$ and the predicted velocity for the entire prediction horizon $N_{p}$ using the equation bellow:

(41) $$\bar{\xi }\left(j\right)=\left[\xi_{0}+\sum_{i=1}^{j}\left(\bar{v}\left(i\right)\;\cdot \;t_{s}\right)\right];\;j=1,2,...,N_{p}$$

$\xi_{0}$ is the current longitudinal position of the subject vehicle and $\bar{v}$ is the predicted nominal velocity. The equation above is substituted into Equation (40) to formulate the generalized constraint equation for $N_{p}$; different constraint equations given below:

(42) $$m_{outer}\left[\xi_{0}+\sum_{i=1}^{j}\left(\bar{v}\left(i\right)\;\cdot \;t_{s}\right)\right]-y\left(j\right)-m_{outer}P_{1x}+P_{1y}<0$$

where the predicted nominal velocity and predicted lateral position can be obtained from the prediction model in MPC. For different values of *j* in (41), Equation (42) represents the outer edge of the virtual straight road constraint, which solely depends on two states of the vehicle (longitudinal velocity and lateral position of the vehicle). Similarly, the line passing through $P_{2}$ with a slope $\psi_{ref}$ forms the right (inner) edge of the virtual straight road. The inner edge of the virtual straight road can be expressed as:

(43) $$y_{inner}=f\left(x_{inner}\right)$$

(44) $$a_{inner}\xi +b_{inner}y+c_{inner}=0$$

where (43) is the equation of the inner lane boundary and (39) is the intersected line perpendicular to the inner lane boundary. Consequently, in the same way as the upper bound, the lower hyperplane can be defined as:

(45) $$m_{inner}\left[\xi_{0}+\sum_{i=1}^{j}\left(\bar{v}\left(i\right)\;\cdot \;t_{s}\right)\right]-y\left(j\right)-m_{inner}P_{4x}+P_{4y}>0$$

To decouple the orientation of the virtual straight road with the subject vehicle’s orientation, the edges are parameterised using $\psi_{ref}$. It is noted that, this technique is suitable to be implemented for different range of curvature of the road. Moreover, it can be ensured that the optimization problem in MPC will always be feasible, and guarantee the convexity on the curve road.

### 5.2. Collision Avoidance Constraints

**Figure 2:**
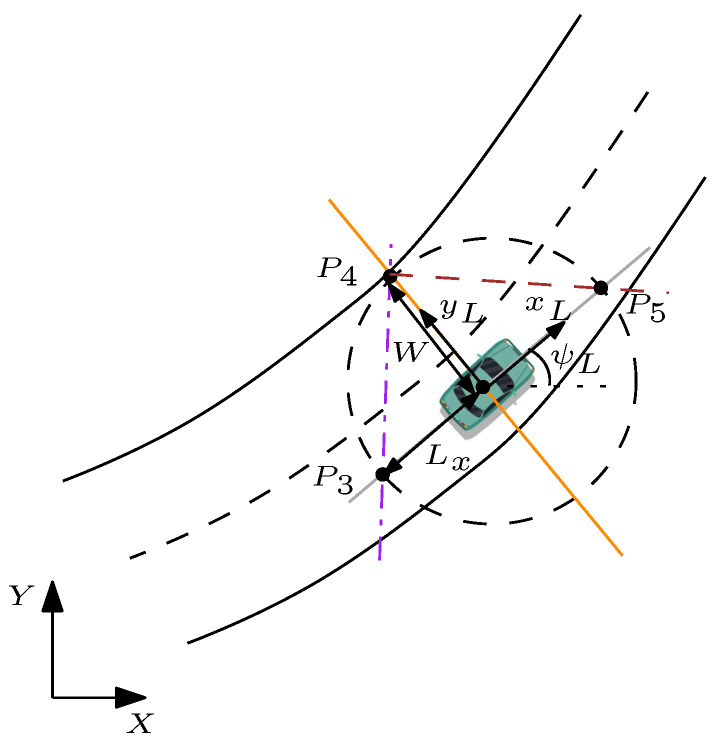
Schematic to construct forward and rear collision avoidance constraints line. Note: $\psi_{L}$ is lead vehicle orientation, $L_{x}$ and *W* indicate the safety distance from the lead vehicle.

As mentioned above the collision avoidance constraints are designed to ensure the subject vehicle evades the obstacle vehicle without any collisions. In literature it is common to design ellipsoids/rectangles around the obstacle to obtain the collision avoidance constraints [30,31]. However, these techniques result in non-convex optimisation problem which limits the uniqueness and feasibility of the solution of the optimisation. In this paper, the collision avoidance constraints are generated using two lines entitled as forward collision avoidance constraints (FCC) and rear collision avoidance constraints (RCC) as shown in Figure 2. This technique is inspired from [24], where the collision avoidance constraints are used for only straight driving conditions. The purpose of generating the FCC is to prevent front end collision while performing a collision-free lane change and the purpose of the RCC is to prevent rear end collision while returning to the original lane. The activation of FCC is when the subject vehicle is behind the lead vehicle while RCC is activated when the subject vehicle crosses the lead vehicle. In this framework, three points ($P_{3},P_{4},P_{5}$) are required to generate collision avoidance constraints. These points can be calculated by intersecting virtual longitudinal and lateral axis of the lead vehicle (grey and yellow lines respectively) with a circle centred at the lead vehicle’s geometrical center, which provides the location of the points $P_{3}$, $P_{4}$, and $P_{5}$. The following subsections explain the procedure for generating collision avoidance constraints.

#### 5.2.1. Forward Collision Avoidance Constraints

**Figure 3:**
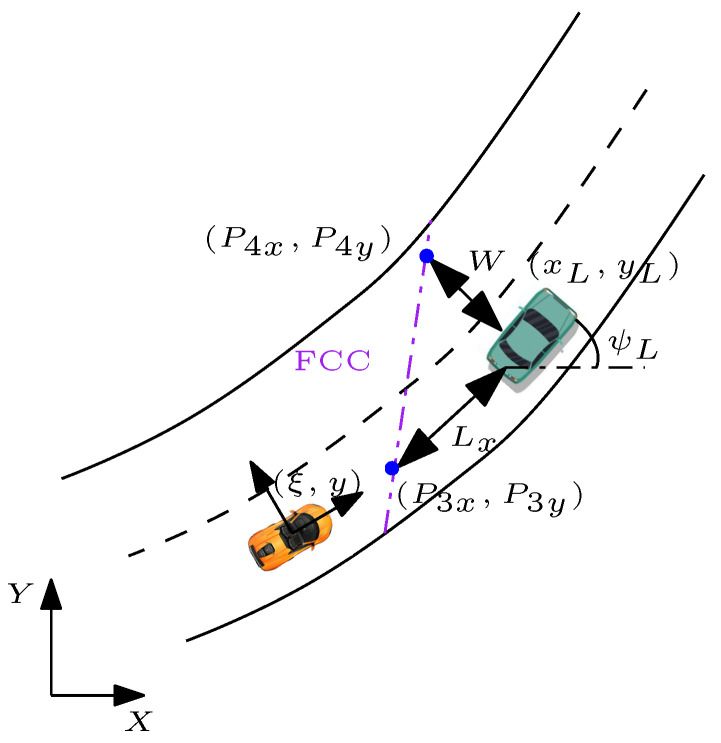
Schematic used to calculate the FCC. Note: orange car—subject vehicle; green car—lead vehicle; red line—RCC; $L_{x}$ and *W* indicate the safety distance from the lead vehicle.

The virtual line on the road segment representing FCC is the line that passes through the points $\left(P_{3x},P_{3y}\right)$ and $\left(P_{4x},P_{4y}\right)$ in Figure 3. Points $\left(P_{3x},P_{3y}\right)$ are obtained as a result of the intersection of the longitudinal axis of the lead vehicle (grey line in Figure 2):

(46) $$a_{Lon}x_{L}+b_{Lon}y_{L}+c_{Lon}=0$$

with a circle centred at the the lead vehicle geometrical centre and radius of $L_{x}+l_{rL}$:

(47) $${\left(x-x_{L}\right)}^{2}+{\left(y-y_{L}\right)}^{2}={\left(L_{x}+l_{rL}\right)}^{2}$$

where $L_{x}$ is the safety distance, which can be defined as $L_{x}=v_{x}t+L_{c}$. The longitudinal velocity of the subject vehicle expressed as $v_{x}$, *t* is the desired time gap of the subject vehicle when approaching the lead vehicle, and finally $L_{c}$ is the lead vehicle length. Moreover, the parameters $l_{rL},a_{Lon},b_{Lon},c_{Lon}$ represent the distance of the centre of gravity of the vehicle to the rear wheels and coefficients of the intersected line, respectively. It is noted that at every time instant of MPC optimization, $L_{x}$ is updated according to the current value of the subject vehicle velocity ($v_{x}$). For generating FCC, another set of points is required $\left(P_{4x},P_{4y}\right)$. These points are generated by means of the intersection of the lateral axis of the lead vehicle (yellow line in Figure 2) with a circle of radius *W*:

(48) $${\left(x-x_{L}\right)}^{2}+{\left(y-y_{L}\right)}^{2}=W^{2}$$

the parameter $W=\frac{1}{2}W_{c}+W_{L}$, where $W_{c}$ is the width of the subject vehicle and $W_{L}$ is the lane width plus the distance from the subject vehicle to the centre line of the road [24]. After defining two sets of points, it would be trivial to find the equation of the line for forward collision avoidance constraints, where the final equation will be:

(49) $$\underset{a_{FCC}}{\underset{︸}{m_{FCC}\;\cdot \;t_{s}}}\left[\sum_{i=1}^{j}\left(\bar{v}\left(i\right)\right)\right]+\left(\underset{b_{FCC}}{\underset{︸}{-1}}\right)y+\underset{c_{FCC}}{\underset{︸}{P_{1y}-m_{FCC}P_{1x}+m_{FCC}\;\cdot \;\xi_{0}}}>0$$

where $m_{FCC}$ is the slope of the forward collision avoidance constraint. The generated FCC divides the (x,y) plane into two regions. Region 1: $a_{FCC}x+b_{FCC}y+c_{FCC}>0$, which is the safe region, and Region 2: $a_{FCC}x+b_{FCC}y+c_{FCC}<0$, which represents the unsafe region. FCC forces the vehicle to be in a safe region while performing a lane change on a curved/straight road. Moreover, (49) represents a linear affine constraint approximation, which can be formulated in QP format.

#### 5.2.2. Rear Collision Avoidance Constraints

**Figure 4:**
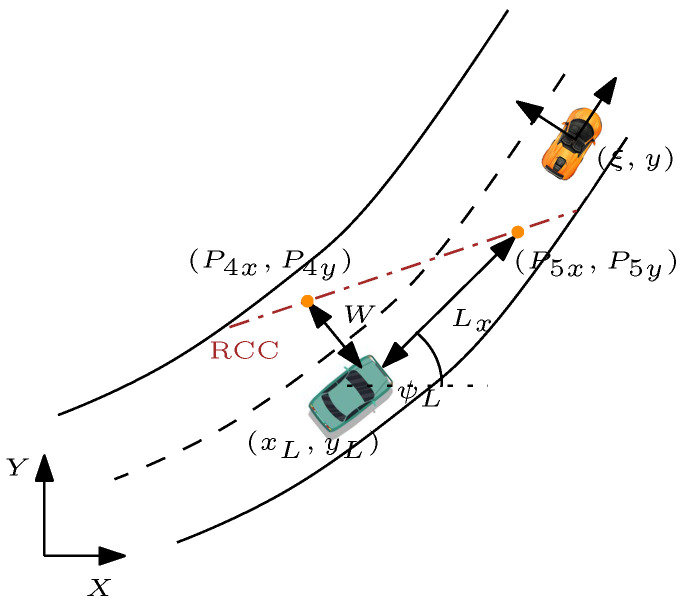
Schematic used to calculate RCC. Note: orange car—subject vehicle; green car—lead vehicle; $L_{x}$ and *W* indicate the safety distance from the lead vehicle.

Similar to FCC, RCC can be generated by following similar steps. The procedure for calculating the intersection point $P_{3}$ is identical to FCC, thus resulting in generating the equation of the RCC line (Figure 4):

(50) $$\underset{a_{RCC}}{\underset{︸}{m_{RCC}\;\cdot \;t_{s}}}\left[\sum_{i=1}^{j}\left(\bar{v}\left(i\right)\right)\right]+\left(\underset{b_{RCC}}{\underset{︸}{-1}}\right)y+\underset{c_{RCC}}{\underset{︸}{P_{3y}-m_{RCC}P_{3x}+m_{RCC}\;\cdot \;\xi_{0}}}<0$$

It is noteworthy that the aforementioned collision avoidance constraints can be adopted for the scenarios where (i) the subject vehicle needs to change a lane while performing the collision avoidance manoeuvre, and (ii) where more traffic members are present on the road that require multiple hyperplanes.

***Remarks***

- One of the benefits of the proposed approach lies in the fact that there is only one parameter to be tuned (desired time gap) and the rest of the parameters are based on the geometry of the lead vehicle.
- The design of the collision avoidance constrains is based on basic mathematical operation; therefore the aforementioned constraints for each traffic vehicle can be generated without any major computation overhead. Thus, it is suitable for real time implementation.
- Reducing a state dimension in the system model helps in bringing down the computation requirement for solving the optimisation problem.

It is noted that by using boundary constraints and the appropriate FCC/RCC based constraints, a convex region representing safe zones on a curved road is generated that forces MPC to plan a safe and collision-free trajectory on the road.

## 6. Trajectory Planning Controller

**Figure 5:**
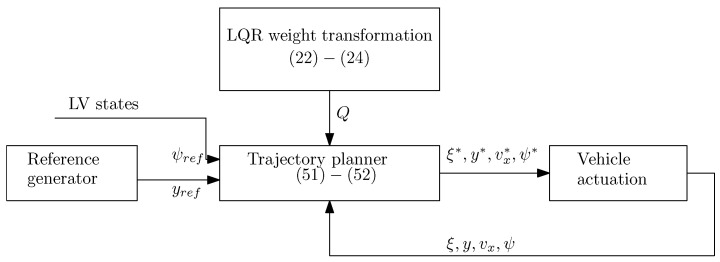
Closed-loop framework for collision avoidance Note: LV indicates lead vehicle.

In general, autonomous highway driving involves motion planning and control of a subject vehicle in order to maintain the velocity and keep the vehicle away from lane boundaries while avoiding possible collisions. The choice of manoeuvre to perform is the result of the decision-making process with the objective of following the desired trajectory $x_{ref},y_{ref}$ (Section 6.1) while respecting physical and design limitations of the subject vehicle. In this section, using the definition of the linear model in (16) and linear system constraints in Figure 5, a constrained linear quadratic optimal control problem is formulated over a prediction horizon $N_{p}$.

### 6.1. Reference Trajectory Generator

The reference trajectory generator provides the longitudinal position $x_{ref}\left(s\right)$ and lateral positions $y_{ref}\left(s\right)$ as well as the orientation ($\psi_{ref}\left(s\right)$) of the road for the vehicle to follow. The generated paths are parametrized by curvature as a function of the distance *s* along the path. The equation of curvature can be formulated as follows:

(51) $$\kappa =\frac{{\dot{x}}_{ref}{\ddot{y}}_{ref}-{\dot{y}}_{ref}{\ddot{x}}_{ref}}{{\left({\dot{x}}_{ref}^{2}+{\dot{y}}_{ref}^{2}\right)}^{\frac{3}{2}}}$$

The reference trajectories are formulated using the clothoids method, which is expressed by Fresnel integrals [32,33]. The trajectories are parametrized by curvature κ as a function of distance *s* along the path as follows:

(52) $$x_{ref}\left(s\right)=\int_{0}^{s}\cos\psi_{ref}\left(x\right)dx$$

(53) $$y_{ref}\left(s\right)=\int_{0}^{s}\sin\psi_{ref}\left(x\right)dx$$

where (52) and (53) are constructed using the polynomial integration method of the Fresnel function applied to clothoid [34], and $\psi_{ref}$ is calculated as follows:

(54) $$\psi_{ref}\left(s\right)=2\tan^{-1}\left(\frac{y_{ref,n}-y_{ref,n-1}}{x_{ref,n}-x_{ref,n-1}}\right)$$

where $\left(x_{ref,n}\right),y_{ref,n}$, n=1...N are the sequence of *N*, the sampled points on the trajectory [35].

### 6.2. Trajectory Planning: MPC

For the trajectory planning using linear MPC, the system states and inputs are subjected to the following state and input constraints:

(55) $$\bar{x}\in \bar{X},u\in U$$

where $\bar{X}=\{\bar{x}\in R^{3}:{\bar{x}}_{min}$≤*x*≤${\bar{x}}_{max}\}\subset R^{3}$ and $U=\{u\in R^{2}:u_{min}$≤*u*≤$u_{max}\}\subset R^{2}$ are states and inputs of the polytope admissible regions (subscripts min and max are the minimum and maximum of the corresponding values). The following cost function is formulated:

(56) $$\begin{array}{c} J=\sum_{k=0}^{N_{p}-1}\left[\right\|{\bar{x}}_{ref}-{\bar{x}}_{t+k|t}{\|}_{Q}^{2}+\|u_{t+k|t}{\|}_{R}^{2}]\\ +\|{\bar{x}}_{ref}-{\bar{x}}_{t+N|t}{\|}_{P}^{2} \end{array}$$

where ${\bar{x}}_{t+k|t}$ is the predicted state trajectory at time t+k obtained by applying the control sequence $U_{t}^{*}={\left[u_{t}^{T},\cdots ,u_{t+N-1}^{T}\right]}^{T}$ to the system (18), starting from an initial state of $\xi_{t|t}$. The parameter $N_{p}\in N^{+}$ is the prediction horizon, whereas $Q\in R^{3\times 3}$, $P\in R^{3\times 3}$ and $R\in R^{2\times 2}$ are weighting matrices. The performance index in (56) consists of the stage cost, input cost and the terminal cost, respectively. The desired state ${\bar{x}}_{ref}$ represents the reference state for the subject vehicle and is defined as ${\bar{x}}_{ref}={\left[y_{ref},v_{xref},\psi_{ref}\right]}^{T}$. It is noted that in this paper, $v_{xref}$ is taken as the initial value of the subject vehicle’s velocity. The following constrained optimization problem, for each sampling time, is formulated as:

(57) $$\min_{U_{t}}J\left(u_{t};\bar{x}\left(t\right),{\bar{x}}_{ref}\right)$$

subject to (18), (42), (45), (49), (50), (55)

In this framework, at every time step, the problem (57), under the constraints (18), (42), (45), (49), (50), (55) are calculated based on the current state x(t), over a shifted time horizon. The optimal control input is calculated as $u^{*}\left(\bar{x}\left(t\right)\right)=U_{t}^{*}\left(0\right)$, which is a solution to the problem (57). As the sets $\bar{X}$ and U are convex, then the MPC problem (57) is solved as a standard QP optimisation problem. All of the steps required to design the proposed MPC controller can be seen in Algorithm 1, and the closed-loop form is illustrated in Figure 5. The following demonstrates the numerical results to show the efficacy of the proposed control strategy.
**Algorithm 1:** Trajectory planning1:*initialize*:2:$v_{des}\leftarrow$ desired longitudinal velocity from user3:**procedure** GENERATE TRAJECTORY4:*top*:5:      Use Equation (3) to create matrix *Q*6:      $\bar{K}\leftarrow$ Ackerman pole placement formula as (4) and calculate $\bar{K}$7:      P← Obtained $\bar{K}$ in (28)–(37) and generate *P*8:      Q← Obtained *P* and substitute (19) in (25)-(27) to calculate *Q*9:*loop*:10:    ${\bar{x}}_{ref}\leftarrow$generate target states as11:    $\bar{x}\leftarrow$current state vector12:    *getRoadBoundaryConstraints* as (38) (39)–(45)13:    *getCollisionAvoidanceConstraints* as (46)–(50)14:    $u^{*}\leftarrow$ solve MPC with generated *Q* from *top* with fixed *R*15:    $x^{*}\leftarrow$ Apply optimal input u(k) as (12)–(15)16:    **if** user request to change $v_{des}$ **then**17:           **goto** *initialize*.18:           **else**19:     **goto** *loop*.

## 7. Numerical Results

In this section, a simulation environment is used to investigate the ability of the proposed trajectory planning architecture to perform a collision avoidance manoeuvre. The scenarios used to perform this are: (1) an obstacle avoidance when the subject vehicle (SV) is 300 m behind the stationary lead vehicle (LV), and travelling at $80\;\mathrm{km}\;h^{-1}$ on a two-lane one-way curved road with a radius of 750 m, (2) a collision avoidance manoeuvre with a moving obstacle under different road surface friction conditions. It is noted that the chosen curved road is approximately equal to the tightest corner allowed in highways [36]. The simulation results for the proposed closed-loop architecture were obtained using the MATLAB IPG CarMaker co-simulation environment.

Moreover, it was assumed that the dimension of the road, lane limits and LVs states are available to the SV, and the initialisation parameters are tabulated in Table 1. Additionally, to evaluate the efficacy of the proposed algorithm, MPC was initialized with a prediction horizon equal to 14 and low time gap (t=0.8) when designing the constraints for imitating more extreme manoeuvres to perform a collision avoidance scenario.

The discrete linear quadratic tracker (6) is used to obtain the proper weighting matrix *Q* in MPC to perform the collision avoidance manoeuvre. The value of the generated matrix *Q* corresponding to the proposed MPC design is Q=diag(1.53,0.023,34.06). It is noted that the simulation results relating to the proposed MPC design was compared with a generic MPC controller where the values of the *Q* matrix have been generated through a trial and error procedure (which will be referred to as manual tuning for the rest of the paper) to demonstrate the superiority of the proposed control algorithm.

It is noted that the choice of manual tuning of $Q_{manual}$ was based on trial and error and the result of many simulations in order to select a set of suitable values to perform trajectory planning with $Q_{manual}=diag\left(5,1e^{-1},230\right)$. Moreover, in both cases the value of the weighting matrix *R* was equally chosen be to *R* = diag($10,9\times 10^{-2}$).

### 7.1. Collision Avoidance with Static Obstacle

**Figure 6:**
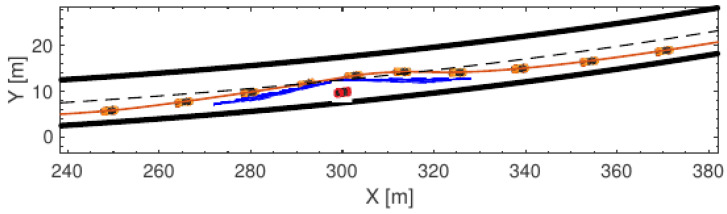
Simulation result: SV and LV (red vehicle) trajectories. Note: FCC and RCC collision avoidance constraints represented as blue dashed lines to provide a safe region between SV and LV.

The result of the obstacle avoidance scenario with the assumption of a high friction surface, using the proposed MPC design, is demonstrated in Figure 6. This figure shows the actual trajectory of the vehicle where the SV starts to change the lane 30 m from the lead vehicle and converges to the original lane shortly after a collision-free path is detected, while staying within the road boundaries. Moreover, it can be seen that the vehicle respects the collision avoidance constraints by maintaining the safety margins from the lead vehicle during the collision avoidance manoeuvre for all sub-manoeuvres. The result in Figure 6 indicates that the proposed structure generated a consistent trajectory with no overshoot while maintaining the safety margins to the lead vehicle during both lane changes.

**Figure 7:**
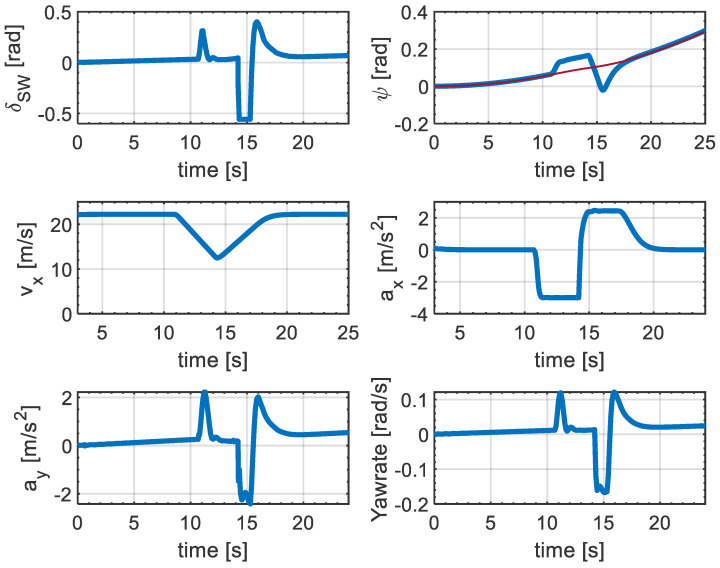
Simulation results: Steering wheel, heading angle (red line indicates the reference heading angle), longitudinal velocity, longitudinal acceleration, lateral acceleration, and yaw-rate for a collision avoidance maneuver.

For further analysis of the performance of the proposed collision avoidance system, the evolution of the states and control inputs of the SV over time is shown in Figure 7. A particular feature of the collision avoidance manoeuvre is that the vehicle starts the manoeuvre close to 11 s where the steering wheel angle for the lateral motion shows a smooth response without having high-frequency oscillation. However, steering action after returning to the original lane is more aggressive and the activation of the input constraints can be observed in this figure. This action suggests that for a successful collision avoidance and accurate reference tracking, large steering action is required on the reverse behaviour, where the SV attempts returning to the original lane. Another key point is the two states of the SV, heading-angle and longitudinal velocity, which demonstrate smooth evolution without having any oscillation during either of the lane changes while maintaining the SV’s pose directionality. It can be seen that the velocity of the vehicle decreases rapidly while the first lane change manoeuvre is being performed. The reverse behaviour (i.e., increasing the velocity while performing the lane change) is visible right after evading the lead vehicle. This is reminiscent of a real-world collision avoidance manoeuvre, where the vehicle decelerate when an obstacle is detected to prevent a possible collision, and accelerates after evading the obstacle, which shows the efficacy of the proposed control structure.

Moreover, the longitudinal acceleration also shows a smooth profile without any oscillation during deceleration and acceleration for both of the lane changes while respecting the system constraints. Similarly, the bottom subplot in Figure 7 demonstrates the lateral acceleration and yaw-rate of the SV, which shows a smooth profile without having high oscillation for both of the lane changes. It is noted that the absolute maximum value of lateral acceleration is $2.3\;m\;s^{-2}$, which demonstrates that the vehicle does not reach its limits of handling. This can be observed by investigating the yaw-rate profile, which the maximum absolute value reaches at about $0.18\;\mathrm{rad}\;s^{-1}$, indicating that the vehicle operates in the linear region of the tire.

**Figure 8:**
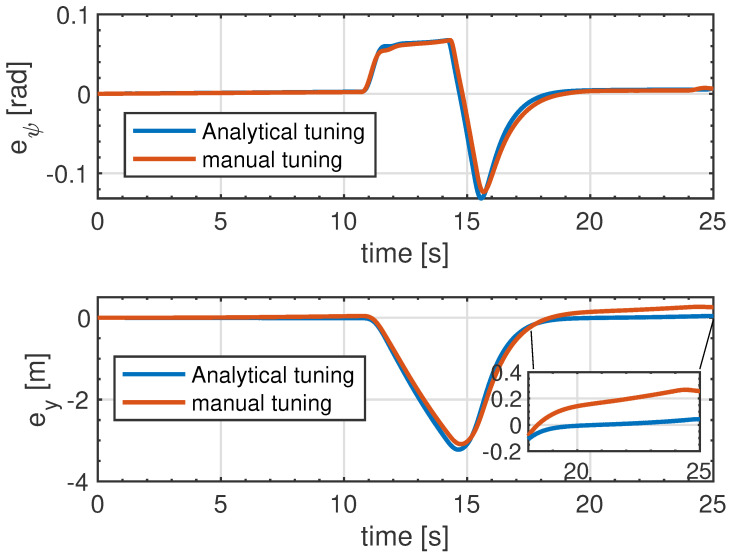
Lateral error $e_{y}$ and heading error $e_{\psi }$ for analytical and manual tuning approach.

To show the need for the proposed MPC design to perform the collision avoidance manoeuvre, we compared the performance of the proposed framework with the MPC design using manual tuning. Figure 8 demonstrates the lateral and heading angle errors for both the analytical tuning and manual tuning of the MPC. In this analysis, the lateral error $e_{y}$ is defined as the lateral distance of the centre of gravity of the vehicle (subject vehicle) from the desired path and $e_{\psi }$ is defined as the difference between the actual orientation of the vehicle and the desired heading angle, which are defined as [37]:

(58a) $$\begin{array}{cc} e_{y} & =\left(y_{CG}-y_{ref}\right)\cos\psi_{ref}-\left(x_{CG}-x_{ref}\right)\sin\psi_{ref} \end{array}$$

(58b) $$\begin{array}{cc} e_{\psi } & =\psi -\psi_{ref} \end{array}$$

The top figure shows the heading angle error, which shows similar behaviour for both the proposed MPC design and manual tuning. It can be seen that the directionality of that vehicle is maintained during the collision avoidance manoeuvre for both cases with minimum discrepancy with the maximum error of 0.15 rad. The bottom figure represents the lateral displacement error for the collision avoidance manoeuvre. The main difference can be observed after performing the collision avoidance manoeuvre, where the error in the analytical tuning strategy almost converges to zero, whereas the conventional tuning reaches about 30 cm. This figure confirms that the proposed strategy is able to successfully maintain the directionality of the vehicle during the collision avoidance manoeuvre while performing better reference tracking with minimum tracking error compared to conventional trial and error tuning.

**Figure 9:**
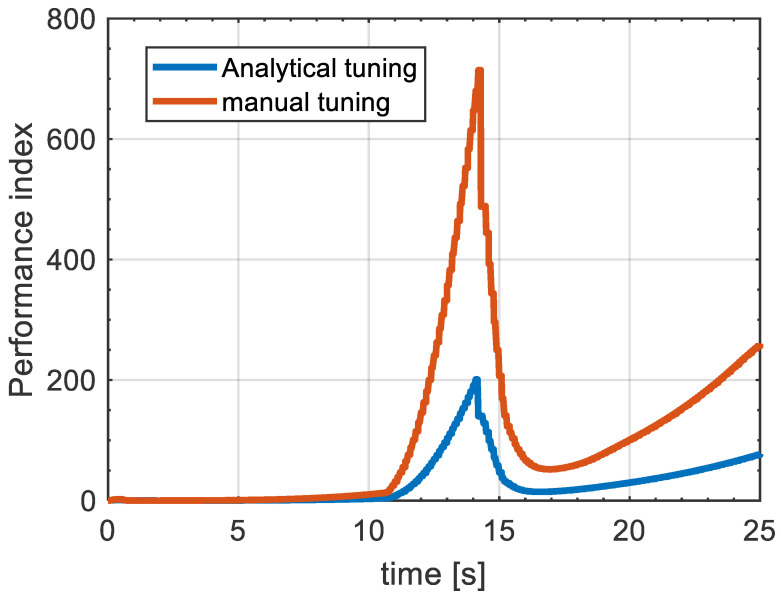
Performance index for both automatic tuning and manual tuning on curved driving condition.

**Figure 10:**
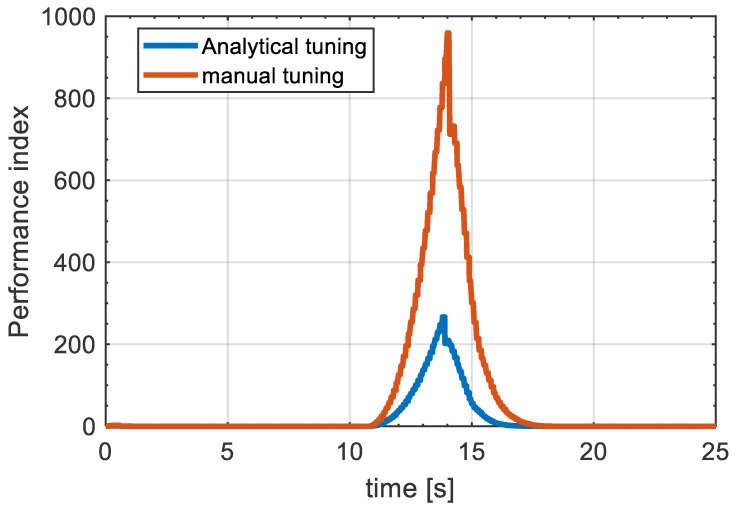
Performance index for both automatic tuning and manual tuning on straight driving condition.

For further illustration of the effectiveness of the proposed MPC design, we compared the performance index value of the proposed technique with the MPC design using manual tuning to investigate the level of optimality between the two procedures. This can be seen in Figure 9, where the performance index (J) indicates the minimized value of the cost function in (56). It can be inferred that the proposed MPC design gives an improvement in J of more than 70% over the manual tuning between 11 s and 15 s, where the performance index is at its maximum, and for the rest of the manoeuvre the performance index for the analytical tuning is still significantly improved compared to the trial and error tuning. For the simulation purposes, the performance index for both methods was analysed for the straight driving scenario. This analysis can be seen in Figure 10, where the performance index is significantly enhanced by the proposed MPC design, which resulted in an improvement close to 80% compared to manual tuning.

### 7.2. Collision Avoidance with Moving Obstacle and Different Surface Friction Conditions

**Figure 11:**
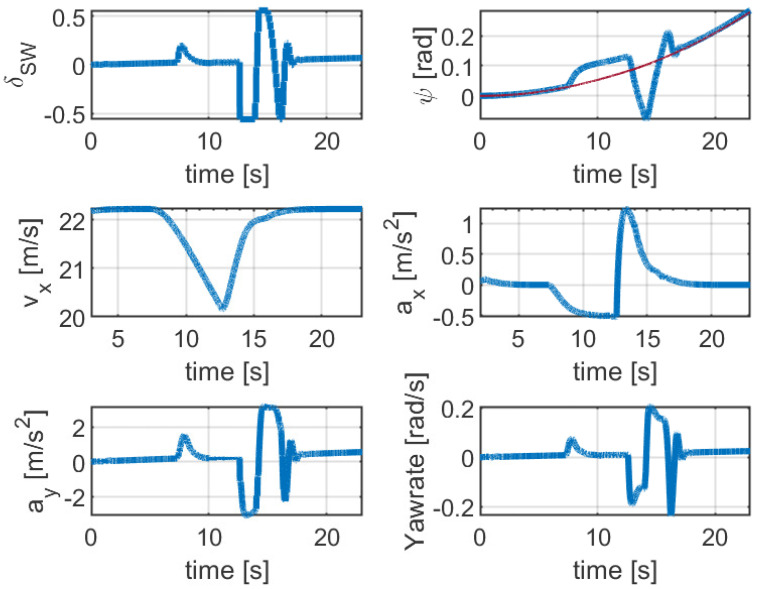
Simulation results: Steering wheel, heading angle (red line indicates the reference heading angle), longitudinal velocity, longitudinal acceleration, lateral acceleration, and yaw-rate for a collision avoidance manoeuvre.

In this section, the efficacy of the proposed algorithm is examined under different values of surface friction. Moreover, the collision avoidance manoeuvre is performed with the presence of a moving vehicle, which resembles a real world driving scenario in the simulation environment. The SV is traveling at $80\;\mathrm{km}\;h^{-1}$ located 300 m behind the LV, and the LV is traveling at $40\;\mathrm{km}\;h^{-1}$ under different values of surface friction conditions ranging from 0.3 to 0.5. Then the system plans the trajectory at each sampling time while applying FCC and RCC constraints based on the position of the subject vehicle and the moving vehicle. Figure 11 illustrates the collision avoidance simulation with an assumption of the lowest friction surface condition (μ=0.3).

It is shown that while the SV vehicle was able to smoothly overtake the moving LV vehicle, it experienced some overshooting when merging back to the original lane. This is due to the sudden and harsh steering actuation of the SV vehicle after performing the first lane change from the LV vehicle. Nevertheless, this amount of actuation is essential in order to maintain the directionality of the vehicle under low surface friction conditions. The results shown in Figure 11 also illustrate that the maximum lateral acceleration reached about $3.5\;m\;s^{-2}$, where the value indicates that the vehicle operates in the linear region of the tire and does not reach its limit of handling. However, this simulation demonstrated some oscillations as the SV vehicle steered back to the original lane, which can be seen in the yaw-rate profile, where its value reached about $0.26\;\mathrm{rad}\;s^{-1}$. Overall, the simulation results indicate that the vehicle respected the collision avoidance constraints and was able to safely converge back to the original lane and follow the desired trajectory on a curved path.

**Figure 12:**
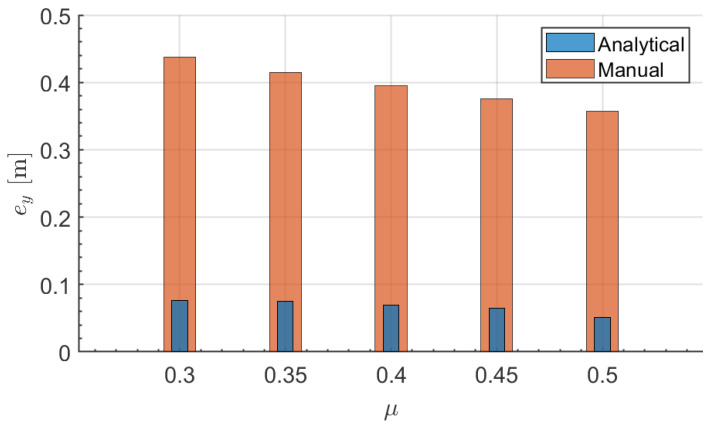
Lateral displacement error over different friction surfaces.

Finally, a parametric analysis was carried out to verify the effectiveness of the proposed framework after performing the collision avoidance manoeuvre. In this analysis, the maximum error between lateral displacement (Figure 12) from the trajectory planner and actual vehicle response was tested in order to investigate the tracking capability of the algorithm under different road surface conditions. It is noted that in this analysis the comparison is made between the proposed analytical MPC and generic MPC controller. The lateral displacement error ($e_{y}$) for analytical tuning increases gradually when lowering the surface friction. This figure indicates a peak deviation of 0.05 m at a 0.3 surface friction, which shows major superiority over manual tuning, which reaches about 0.44 m with the same friction value. This trend is similar for the rest of the friction values, which demonstrates the effectiveness of the proposed control algorithm to improve vehicle handling while performing the collision avoidance manoeuvre.

## 8. Conclusions

In this paper, we have presented an MPC-based collision avoidance framework by means of an analytical tuning methodology. The main contribution of this paper lies in the derivation of the transformation of a discrete time LQR weighting matrix *Q* to generate the MPC weighting matrix. The importance of analytical tuning was investigated next, with results showing that it not only improves the tracking error, but can also minimize the performance index compared to manual tuning of MPC. Moreover, the analytical tuning design was then tested under various friction conditions to investigate the efficacy of the proposed algorithm. The results show that while the manual tuning of MPC is effective, the analytical tuning can achieve better performance that is close to the optimal solution while performing a collision avoidance manoeuvre. The numerical results obtained in the Simulink/IPG Carmaker co-simulation environment indicated that the proposed technique fulfills the safety considerations and generates a feasible and collision-free trajectory on a curved road. This work offers a number of suggestions for future research directions. (1) The weighting matrix calculation was based on the gain matrix $\bar{K}$ in (4), which needed to be specified such that the closed-loop poles remain inside of the unit circle. However, the selection of the poles was chosen using Ackerman’s pole placement method. This approach can be extended by means of reinforcement learning in future research. In this way, the position of the poles inside of the unit circle can be adjusted depending on different operating conditions, such as variations in surface friction. (2) A path tracking controller can be integrated as a lower-level controller to capture the vehicle dynamics to improve the stability and handling of the vehicle in extreme driving conditions.

## Figures and Tables

**Table 1:** Design Parameters.

Symbols	Value	Value
	Road Geometry	
$N_{lanes}$	2	-
$w_{lane}$	5	m
	Subject Vehicle	
$l_{f}$	1.144	m
$l_{r}$	1.2060	m
	Lead Vehicle	
$l_{Lv,l}$	4.1	m
$l_{Lv,w}$	1.7	m
	Controller Parameters	
$t_{s}$	0.1	s
$N_{p}$	14	-
closed-loop poles	($5e^{-1},0.6,0.95$)	-
	System	
X	−[500, −11.1,1.5] ≤ *x* ≤ [500,27.7778,1.5]	-
U	−[0.0698, 3] ≤ *u* ≤ [0.0698,3]	-

## Data Availability

Not applicable.

## References

[1] Mohammadi A., Asadi H., Mohamed S., Nelson K., Nahavandi S. (2019). Multiobjective and Interactive Genetic Algorithms for Weight Tuning of a Model Predictive Control-Based Motion Cueing Algorithm. IEEE Trans. Cybern..

[2] Gray A., Gao Y., Lin T., Hedrick J.K., Tseng H.E., Borrelli F. Predictive control for agile semi-autonomous ground vehicles using motion primitives. Proceedings of the 2012 American Control Conference.

[3] Dixit S., Montanaro U., Fallah S., Dianati M., Oxtoby D., Mizutani T., Mouzakitis A. Trajectory Planning for Autonomous High-Speed Overtaking using MPC with Terminal Set Constraints. Proceedings of the 2018 21st International Conference on Intelligent Transportation Systems.

[4] Fontes M., Martins A.F., Odloak D. (2019). An automatic tuning method for model predictive control strategies. Proc. Am. Control Conf..

[5] Julian B.-G. (2017). Tuning for MPC Controllers. The Role of Population Games in the Design of Optimization-Based Controllers.

[6] Cairano S., Bemporad A. (2010). Model Predictive Control Tuning by Controller Matching. IEEE Trans. Autom. Control.

[7] Shah G., Engell S. Tuning MPC for desired closed-loop performance for SISO systems. Proceedings of the 18th Mediterranean Conference on Control and Automation, MED’10-Conference Proceedings.

[8] Clarke D.W., Mohtadi C., Tuffs P.S. (1987). Generalized predictive control-Part I. The basic algorithm. Automatica.

[9] Klopot T., Skupin P., Metzger M., Grelewicz P. (2018). Tuning strategy for dynamic matrix control with reduced horizons. ISA Trans..

[10] Shen C., Gonzalez Y., Chen L., Jiang S., Jia X. (2018). Intelligent Parameter Tuning in Optimization-Based Iterative CT Reconstruction via Deep Reinforcement Learning. IEEE Trans. Med. Imaging.

[11] Qi Z., Shi Q., Zhang H. (2019). Tuning of digital PID controllers using particle swarm optimization algorithm for a CAN-based DC motor subject to stochastic delays. IEEE Trans. Ind. Electron..

[12] Grosso J.M., Ocampo-Martínez C., Puig V. (2013). Learning-based tuning of supervisory model predictive control for drinking water networks. Eng. Appl. Artif. Intell..

[13] Lee J.H., van der Svrcek W.Y., Young B.R. (2007). A tuning algorithm for model predictive controllers based on geneticalgorithms and fuzzy decision makingtle. ISA Trans..

[14] Jorge L. (2010). Garriga and Masoud Soroush. Model Predictive Control Tuning Methods: A Review. Ind. Eng. Chem..

[15] Gutiérrez-Urquídez R.C., Valencia-Palomoa G., Rodríguez-Elias O.M., Trujillob L. (2015). Systematic selection of tuning parameters for efficient predictive controllers using a multiobjective evolutionary algorithm. Appl. Soft Comput..

[16] Qi Z., Shi Q., Zhang H. (2019). Inverse optimal control for discrete-time finite-horizon Linear Quadratic Regulators. Automatica.

[17] Willems J., Van De Voorde H. (1977). Inverse optimal control problem for linear discrete-time systems. Electron. Lett..

[18] Luo W., Chu Y.C., Ling K.V. (2005). Inverse optimal adaptive control for attitude tracking of spacecraft. IEEE Trans. Autom. Control.

[19] Halder K., Das S., Gupta A. (2019). Transformation of LQR Weights for Discretization Invariant Performance of PI/PID Dominant Pole Placement Controllers. Robotica.

[20] Kiumarsi-Khomartash B., Frank L., Naghibi-Sistani L., Mohammad B., Karimpour A. Optimal tracking control for linear discrete-time systems using reinforcement learning. Proceedings of the 52nd IEEE Conference on Decision and Control.

[21] Franze G., Lucia W. (2016). A Receding Horizon Control Strategy for Autonomous Vehicles in Dynamic Environments. IEEE Trans. Control Syst. Technol..

[22] Gao Y., Gray A., Frasch J., Lin T., Tseng E., Hedrick J.K., Borrelli F. Spatial Predictive Control for Agile Semi-Autonomous Ground Vehicles. Proceedings of the 11th International Symposium on Advanced Vehicle Control.

[23] Karlsson J., Murgovski N., Sjöberg J. Temporal vs. Spatial formulation of autonomous overtaking algorithms. Proceedings of the 2016 IEEE 19th International Conference on Intelligent Transportation Systems.

[24] Nilsson J., Falcone P., Ali M., Sjöberg J. (2015). Receding horizon maneuver generation for automated highway driving. Control Eng. Pract..

[25] Kong J., Pfeiffer M., Schildbach G., Borrelli F. Kinematic and Dynamic Vehicle Models for Autonomous Driving Control Design. Proceedings of the 2015 IEEE Intelligent Vehicles Symposium.

[26] Liu C., Lee S., Varnhagen S., Tseng H.E. Path planning for autonomous vehicles using model predictive control. Proceedings of the 2017 IEEE Intelligent Vehicles Symposium.

[27] Gutjahr B., Gröll L., Werling M. (2017). Lateral Vehicle Trajectory Optimization Using Constrained Linear Time-Varying MPC. IEEE Trans. Intell. Transp. Syst..

[28] Mad D., Philippsen R., Eidehall A., Dahl K. On Path Planning Methods for Automotive Collision Avoidance. Proceedings of the 2013 IEEE Intelligent Vehicles Symposium.

[29] Nilsson J., Gao Y., Carvalho A., Borrelli F. (2014). Manoeuvre generation and control for automated highway driving. IIFAC Proc. Vol..

[30] Gao Y., Gray A., Carvalho A., Tseng H.E., Borrelli F. Robust nonlinear predictive control for semiautonomous ground vehicles. Proceedings of the 2014 American Control Conference.

[31] Plessen M., Lima P., Martensson J., Bemporad A., Wahlberg B. Trajectory planning under vehicle dimension constraints using sequential linear programming. Proceedings of the 2017 IEEE 20th International Conference on Intelligent Transportation Systems.

[32] Scheuer A., Fraichard T. (1997). Collision-free and continuous-curvature path planning for car-like robots. Proc. IEEE Int. Conf. Robot. Autom..

[33] Shin D.H., Singh S. (1992). Path Generation for a Robot Vehicle Using Composite Clothoid Segments. IFAC Proc. Vol..

[34] The Practice of Clothoid Connections on Road Interchanges. http://precismultipla.altervista.org/ESU2/chap08.htm.

[35] Funke J. (2015). Collision Avoidance Up to the Handling Limits for Autonomous Vehicles. Ph.D. Thesis.

[36] Schmeitz A., Zegers J., Ploeg J., Alirezaei M. Towards a generic lateral control concept for cooperative automated driving theoretical and experimental evaluation. Proceedings of the 5th IEEE International Conference on Models and Technologies for Intelligent Transportation Systems, MT-ITS 2017-Proceedings.

[37] Chatzikomis C., Sorniotti A., Gruber P., Zanchetta M., Willans D. (2018). Comparison of Path Tracking and Torque-Vectoring Controllers for Autonomous Electric Vehicles. IEEE.

